# Ocular ischemic syndrome secondary to cerebral aneurysms

**DOI:** 10.1016/j.ajoc.2024.102214

**Published:** 2024-10-31

**Authors:** Landon J. Rohowetz, Patrick Staropoli, Natasha F.S. da Cruz, Carlos Mendoza, Robert M. Starke, Jacques J. Morcos, Audina M. Berrocal

**Affiliations:** a Department of Ophthalmology, Bascom Palmer Eye Institute, 900 NW 17th St., Miami, FL, USA; b Department of Neurological Surgery, University of Miami, Jackson Health System, 1095 Northwest 14th Ter., Miami, FL, 33136, USA

**Keywords:** Cerebral aneurysm, Hemifacial microsomia, Neovascularization, Ocular ischemic syndrome

## Abstract

**Purpose:**

To describe the clinical findings in an 11-year-old male with a history of hemifacial microsomia presenting with ocular ischemic syndrome secondary to large cerebral aneurysms.

**Observations:**

An 11-year-old male with a history of hemifacial microsomia presented to the Bascom Palmer Eye Institute Emergency Department complaining of nausea, diarrhea, headache, and decreased vision in the left eye. Visual acuity was light perception in the left eye and intraocular pressure was within normal limits. Gonioscopy revealed the presence of diffuse neovascularization of the angle. Posterior segment examination revealed mild vitreous hemorrhage, optic disc pallor, preretinal hemorrhage, generalized arteriolar narrowing, retinal microaneurysms, and abnormal arteriovenous communications with branching retinal vessels. Fluorescein angiography demonstrated patchy and delayed choroidal filling, a prolonged venous filling time, arteriolar attenuation, and vascular staining consistent with ocular ischemic syndrome. Magnetic resonance angiography was obtained which revealed large left internal carotid and anterior cerebral artery aneurysms. The patient underwent successful cerebral revascularization via bypass, ligation, clipping, and coiling procedures. At postoperative year 1, there was no evidence of ocular neovascularization and visual acuity remained light perception.

**Conclusion and Importance:**

Ocular ischemic syndrome is uncommon in children but may occur with any cause of ocular hypoperfusion. Hemifacial microsomia is a rare congenital disorder of craniofacial development caused by a vascular event in utero affecting the first and second branchial arches. This case demonstrates a rare cause of ocular ischemic syndrome and illustrates the potential for the development of clinically significant vascular abnormalities in patients with disorders of craniofacial development.

## Highlights

• Ocular ischemic syndrome (OIS) is characterized by hypoperfusion of the ocular tissues.
• OIS is most commonly caused by carotid artery stenosis.
• Hemifacial microsomia is a disorder affecting the first and second branchial arches.
• A child with hemifacial microsomia presented with OIS due to cerebral aneurysms.
• Cerebral aneurysm is rare in hemifacial microsomia and an uncommon cause of OIS.

## 1 Introduction

Ocular ischemic syndrome (OIS) is caused by hypoperfusion of the eye and typically occurs secondary to carotid artery stenosis due to atherosclerosis.^1^ The condition is most commonly seen in older adults and is rare before 50 years of age.^2^ OIS is frequently the first sign of underlying vascular disease and may precede the development of more severe and potentially lethal complications such as cerebrovascular accident and myocardial infarction if not urgently addressed.^3^^,^^4^ Rare causes of OIS include aortic arch syndrome and vasculitides such as giant cell and Takayasu arteritis.^5^, ^6^, ^7^ In the current report, we describe a child with a history of hemifacial microsomia presenting with OIS secondary to cerebral aneurysms.

## 2 Case report

An 11-year-old male presented to the general emergency department complaining of a headache. He had a history of left hemifacial microsomia and associated left-sided facial weakness. The patient complained of nausea, vomiting, and diarrhea beginning 5 days prior to presentation. This was followed by a headache the subsequent night that the patient described as a throbbing sensation over his left eye. In the emergency department, the patient appeared in no acute distress and was afebrile with normal vital signs. Complete blood count and comprehensive metabolic panel were unremarkable. Neurological examination revealed no acute findings. The patient's headache resolved with the administration of intravenous ketorolac, ondansetron, diphenhydramine, and normal saline. He was discharged home with instructions to follow up with his primary care physician in 1–2 days.

**Fig. 1:**
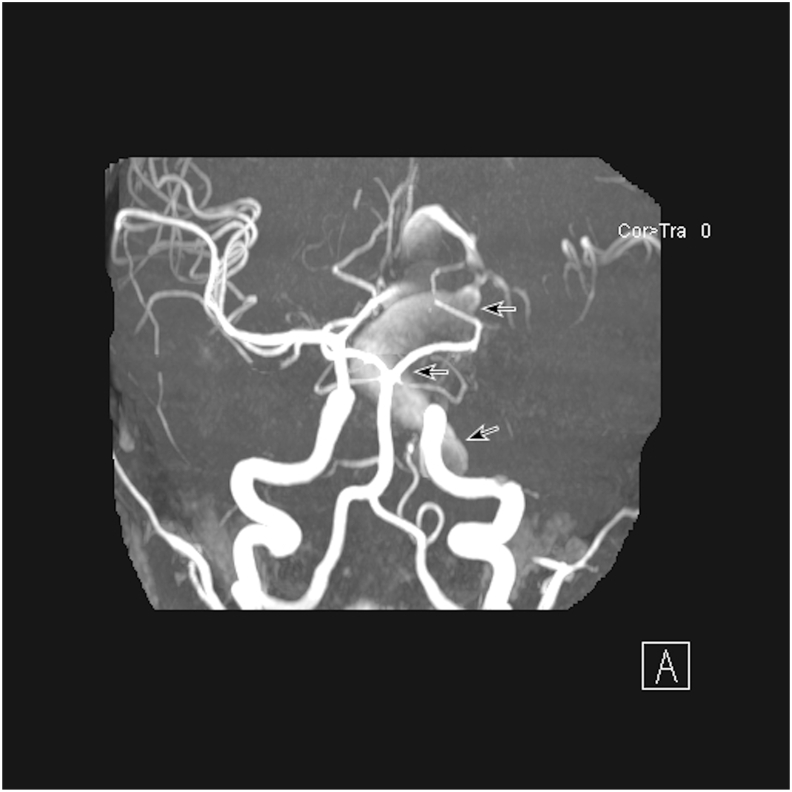
Magnetic resonance angiography at diagnosis Magnetic resonance angiography of the brain of an 11-year-old male with a history of hemifacial microsomia and poor visual acuity in the left eye with ocular neovascularization demonstrating large left internal carotid and anterior cerebral artery aneurysms (arrows).

Three days later, the patient presented to the Bascom Palmer Eye Institute Emergency Department complaining of decreased visual acuity in the left eye. The decreased vision began shortly after the aforementioned emergency department visit. He had seen an ophthalmologist the day prior to presentation who noted optic disc pallor in the left eye. Visual acuity with correction was 20/20 in the right eye and light perception with a 3+ relative afferent pupillary defect in the left eye. Intraocular pressures were 13 and 14 in the right and left eyes, respectively. Color vision was normal in the right eye and unable to be assessed in the left due to inadequate visual acuity. Perceived brightness with a muscle light shown in the left eye was 1–2 out of 10 compared to 10 out of 10 in the right. Extraocular movements were full without pain in both eyes. Anterior segment examination was unremarkable in both eyes other than prominent corneal nerves. Gonioscopy of the left eye was open to ciliary body band with diffuse neovascularization of the angle. Posterior segment examination of the right eye was normal with a small optic cup. The left eye demonstrated mild vitreous hemorrhage, optic disc pallor with a small optic cup, preretinal hemorrhage in the inferior macula, generalized arteriolar narrowing, a fibrotic aneurysm in the supratemporal periphery with adjacent dot-blot hemorrhages, microaneurysms in the macula, and abnormal arteriovenous communications with branching vessels. Given the history of left-sided hemifacial microsomia with new-onset neovascularization, the patient was transferred to the general hospital. Coagulation profile including prothrombin time, partial thromboplastin time, and fibrinogen level was unremarkable. Magnetic resonance imaging and magnetic resonance angiography were performed which revealed a giant fusiform left internal carotid artery (ICA) aneurysm with a separate partially thrombosed anterior cerebral artery aneurysm (ACA; Fig. 1). The patient underwent successful bypass from the left common carotid artery to the left frontal M2 branch of the middle cerebral artery, ligation of the left cervical ICA, and clipping of the left A1 segment of the ACA. There was no room to trap the distal intracranial ICA due to involvement of the anterior choroidal artery, and therefore the final therapeutic treatment constitutes bypass and “flow reversal” into the fusiform left ICA aneurysm. The fusiform ACA aneurysm was coil occluded via the right A1 segment of the ACA.

**Fig. 2:**
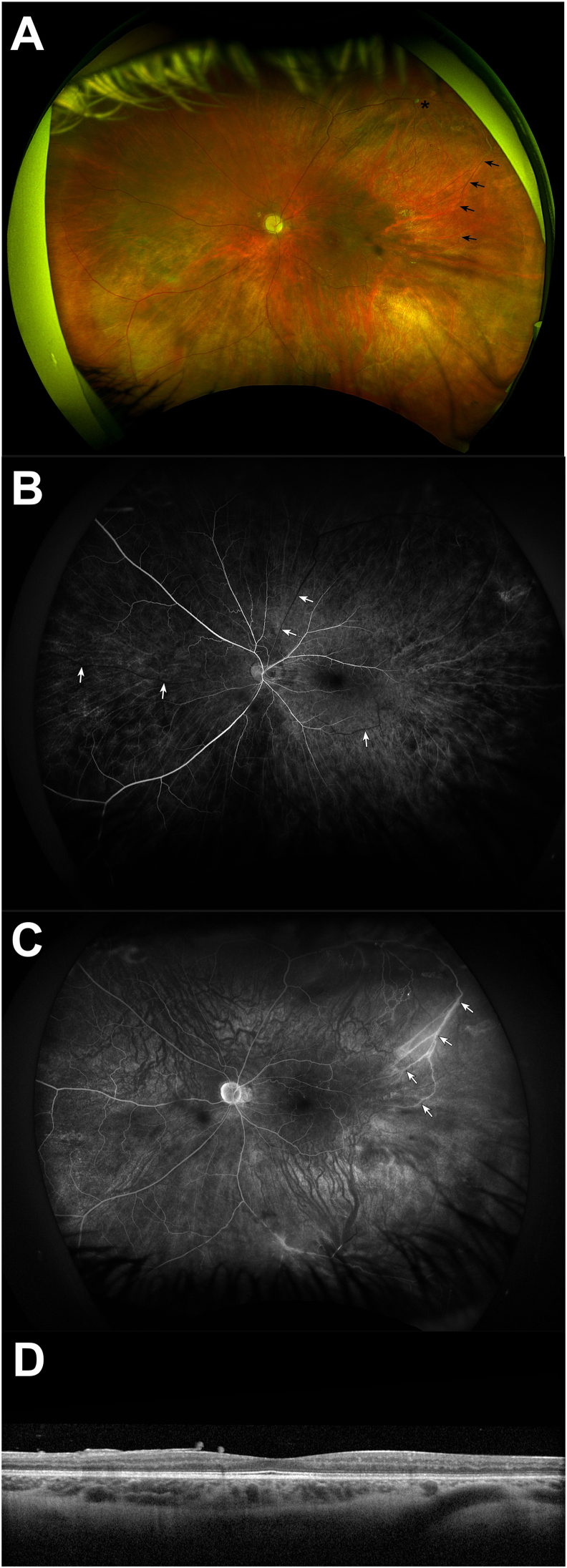
Ophthalmic imaging of the left eye An 11-year-old male with ocular ischemic syndrome due to cerebral aneurysms. Fundus photography (A) demonstrates resolving vitreous hemorrhage, optic disc pallor with a small optic cup, generalized arteriolar narrowing, a fibrotic aneurysm in the supratemporal periphery (asterisk) with adjacent dot-blot hemorrhages, microaneurysms in the macula, and arteriovenous communications with abnormal branching vessels (arrows). Early phase fluorescein angiography at 29 seconds (B) demonstrates patchy and delayed choroidal filling, few small microaneurysms in the macula, arteriolar narrowing, and delayed venous filling (arrows). Late phase fluorescein angiography at 11 minutes (C) demonstrates peripheral branching vessels with abnormal arteriovenous communications (arrows) and associated staining. Optical coherence tomography (D) demonstrates moderate diffuse retinal atrophy, severe ganglion cell layer thinning, and mild vitreous opacities.

The patient returned to the ophthalmology clinic 2 weeks later. Visual acuity was still light perception. There was trace persistent neovascularization of the angle. Intraocular pressure was normal at 16 mm Hg. Examination demonstrated resolved preretinal and resolving vitreous hemorrhage in addition to the aforementioned examination findings (Fig. 2A). Fluorescein angiography demonstrated patchy and delayed choroidal filling, arteriolar narrowing, prolonged arteriovenous filling time, few small macular microaneurysms, and peripheral branching vessels with associated staining (Fig. 2B and C). Optical coherence tomography demonstrated moderate diffuse retinal atrophy (Fig. 2D) with severe thinning of the retinal nerve fiber and ganglion cell layers.

**Fig. 3:**
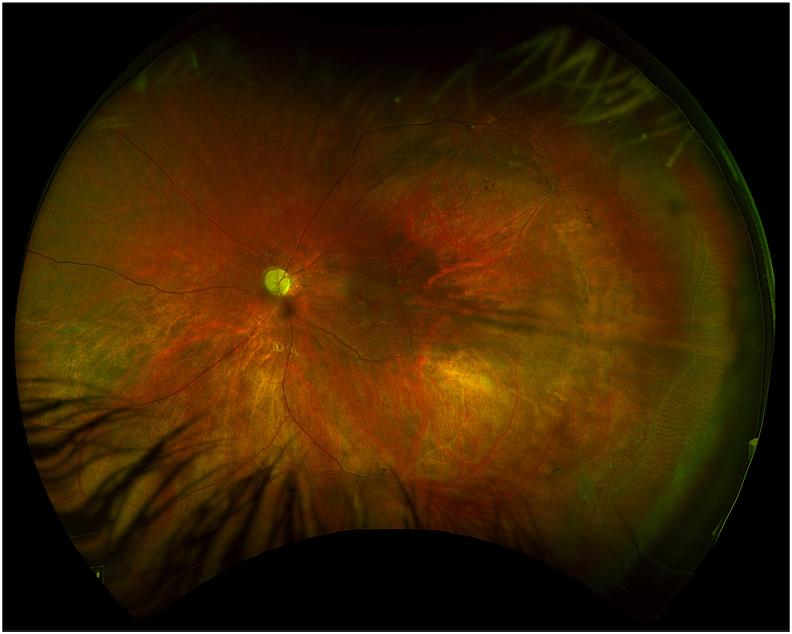
Fundus photography of the left eye at postoperative year 1 Fundus photography 1 year after cerebral revascularization demonstrating resolution of vitreous hemorrhage with persistence of optic disc pallor and central and peripheral vascular abnormalities.

**Fig. 4:**
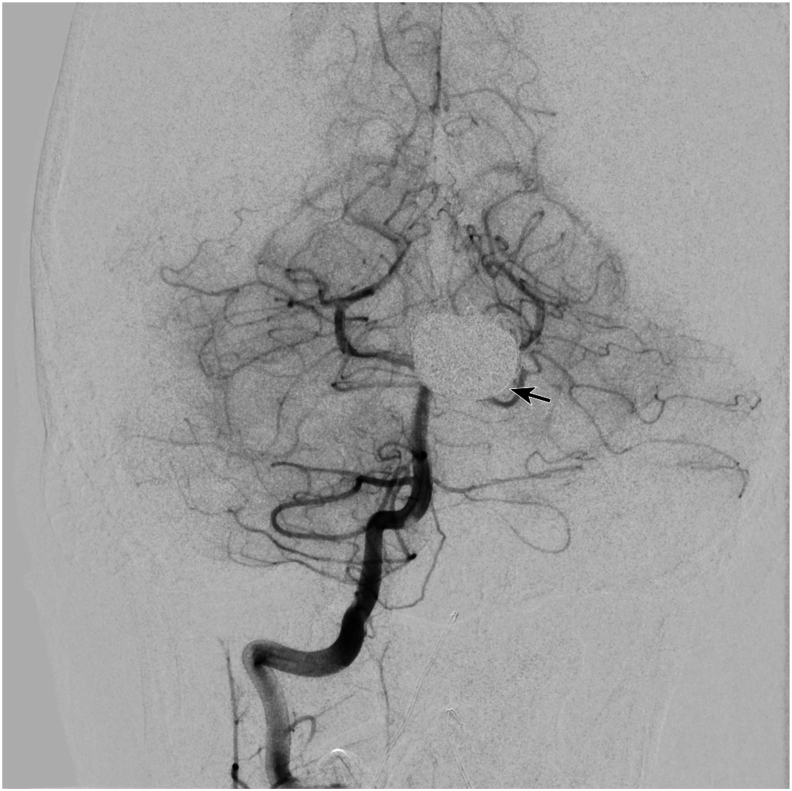
Cerebral angiogram at postoperative year 1 Cerebral angiogram at postoperative year 1 demonstrating bypass patency and no residual filling of the left internal carotid or anterior cerebral artery aneurysms. Coiling can be seen in the A1 segment of the anterior cerebral artery aneurysm (arrow).

At postoperative year 1, visual acuity was still light perception. The neovascularization of the angle had resolved and examination was otherwise stable (Fig. 3). Diagnostic cerebral angiography demonstrated patency of the bypass and no residual filling of the aneurysms (Fig. 4).

## 3 Discussion

OIS is caused by hypoperfusion of ocular tissues.^1^ Patients typically present with signs of vision loss and anterior or posterior segment inflammation.^1^ The most frequent cause is carotid artery stenosis due to atherosclerosis and the condition is rare in children.^2^ In this report, we describe an 11-year-old patient with a history of hemifacial microsomia presenting with OIS secondary to large cerebral aneurysms.

Examination findings in OIS may include a combination of iris neovascularization, neovascular glaucoma, anterior chamber inflammation, retinal arteriolar narrowing, venular dilatation, retinal hemorrhages, and retinal neovascularization, most of which were seen in the current patient.^2^ The current patient also presented with ischemic optic neuropathy as evidenced by optic nerve pallor on examination and diminished retinal nerve fiber and ganglion cell layers on optical coherence tomography. Indeed, anterior ischemic neuropathy has been reported in 2–18 % of eyes with OIS and may portend a poor visual prognosis.^2^^,^^8^, ^9^, ^10^

Although carotid artery stenosis is the most common cause of OIS, other rare causes include giant cell arteritis, Takayasu arteritis, aortic arch syndrome, neurofibromatosis, moyamoya disease, radiation therapy, and carotid artery dissection.^5^^,^^6^^,^^11^, ^12^, ^13^, ^14^, ^15^, ^16^, ^17^ Patients with OIS demonstrate decreased blood flow in the retrobulbar arteries and a reversal of blood flow through the ophthalmic artery.^1^^,^^18^ Several researchers believe the ophthalmic artery acts as a steal artery shunting blood from the ophthalmic circulation to the low pressure intracranial system leading to ischemia of the ocular tissues.^1^^,^^19^ In the current patient, hypoperfusion of the ophthalmic artery was caused by large cerebral aneurysms.

Hemifacial microsomia is a rare congenital malformation of the first and second branchial arches and is characterized by variable unilateral hypoplasia of the facial skeleton and surrounding soft tissue.^20^, ^21^, ^22^ Although a variety of theories exist regarding the etiology of the malformations, they are thought to arise as a result of an abnormality or disruption in the blood supply to the developing craniofacial region, particularly a stapedial artery aneurysm, during early embryogenesis.^21^^,^^23^ This disruption in blood supply is believed to be a transient event with patients carrying no known predisposition to developing cerebrovascular abnormalities in later life.^24^ However, other disorders of craniofacial development such as Goldenhar and Treacher-Collins syndrome have been associated with the development of cerebral vascular abnormalities including ICA aneurysms.^25^^,^^26^ As such, screening for cerebrovascular abnormalities in patients with congenital craniofacial malformations may be considered in select cases.

The visual prognosis in OIS is guarded and highly dependent on visual function at diagnosis.^4^^,^^27^ Sivalingam and colleagues demonstrated the presence of counting fingers vision or worse in the majority of patients in their study with OIS 1 year after diagnosis.^27^ Furthermore, Mizener and colleagues demonstrated that all eyes in their cohort of OIS patients with visual acuity of 20/400 or worse at presentation had counting fingers or worse vision at last follow-up.^4^ There were no differences in visual acuity at last follow-up in eyes that underwent carotid endarterectomy and those that did not.^4^ Indeed, the patient in the current report presented with light perception vision likely secondary to retinal and optic atrophy with no subsequent visual improvement despite successful cerebral revascularization.

## 4 Conclusions

OIS is caused by hypoperfusion of the ocular tissues and is most commonly due to carotid artery stenosis.^1^^,^^2^ Hemifacial microsomia is a rare disorder of unilateral craniofacial development thought to be caused by a vascular event in utero affecting the first and second branchial arches.^20^, ^21^, ^22^ In this case report, we describe a child with a history of hemifacial microsomia presenting with OIS secondary to large cerebral aneurysms. This case illustrates a rare cause of OIS and demonstrates the potential for the development of clinically significant vascular abnormalities in patients with disorders of craniofacial development.

## CRediT authorship contribution statement

**Landon J. Rohowetz:** Writing – review & editing, Writing – original draft, Investigation, Data curation. **Patrick Staropoli:** Writing – review & editing, Investigation, Data curation. **Natasha F.S. da Cruz:** Writing – review & editing, Investigation, Data curation. **Carlos Mendoza:** Investigation, Data curation. **Robert M. Starke:** Data curation, Writing – review & editing. **Jacques J. Morcos:** Data curation, Writing – review & editing. **Audina M. Berrocal:** Writing – review & editing, Supervision, Investigation, Data curation, Conceptualization.

## Patient consent

The patient(s)/patient's legal guardian consented to publication of the case in writing.

## Authorship

All authors attest that they meet the current ICMJE criteria for Authorship.

## Funding

Research to Prevent Blindness-Unrestricted Grant to BPEI (GR004596-1; New York, NY). The funding sources had no role in study design, data collection, analysis and interpretation of data, writing of the report, or in the decision to submit the article for publication.

## Declaration of competing interest

The authors declare the following financial interests/personal relationships which may be considered as potential competing interests: AMB is a consultant for Alcon, Allergan, Zeiss, Dutch Ophthalmic Research Center, Novartis, ProQR, and Oculus. The following authors have no financial disclosures: LJR, PS, NFSC, CM, RMS, JJM.
